# Supplementary material for: Metformin Mitigates Nickel-Elicited Angiopoietin-Like Protein 4 Expression via HIF-1α for Lung Tumorigenesis
Source: Int J Mol Sci. 2020 Jan 17;21(2):619. doi: 10.3390/ijms21020619 (PMC7014330; doi:10.3390/ijms21020619)
Supplement: Supplementary file 1 [file ijms-21-00619-s001.pdf]

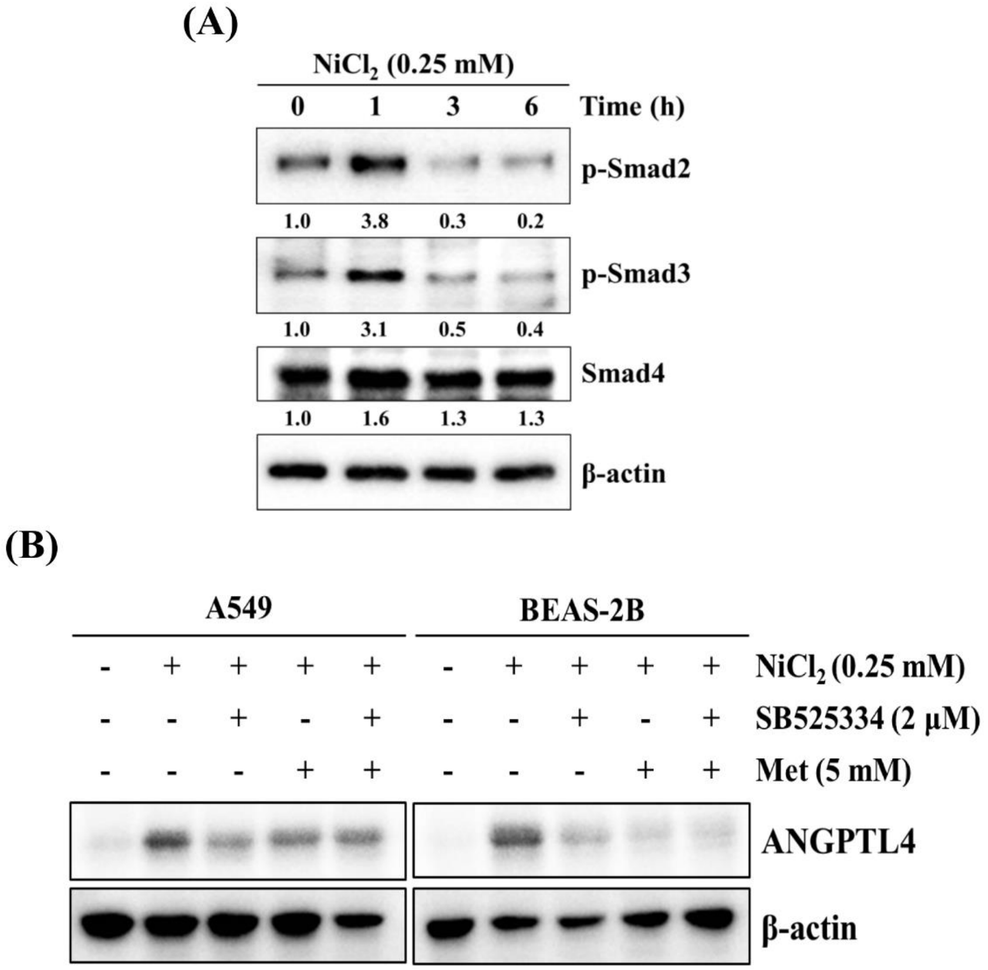

Supplementary FigureS1. $NiCl_2$ increases the expression of ANGPTL4 via TGF-β signaling pathway. (A) BEAS-2B cells were treated with 0.25 mM $NiCl_2$ for 0, 1, 3 and 6 h, The protein levels of p-smad2, p-samd3 and samd4 were analyzed by Western blot. (B) A549 and BEAS-2B cells were treated with SB525334 (2μM) and/or metformin (5 mM) underling $NiCl_2$ exposure for 24 h, the protein expression of ANGPTL4 was performed. β-actin was used as the internal control.

Supplemental Table S1. Primers list for expression analysis.

Analysis	Gene symbol	Forward (5′-3′)	Reverse (5′-3′)
**Q-PCR**	ANGPTL4	CTCCACTTGGGACCAGGATC A	ATGGCTGCAGGTGCCAAAC
	HIF-1α	TAGCCGAGGAAGAACTATGAAC	CACACTGAGGTTGGTTACTG TTG
	β-actin	TCATCACCATTGGCAATGAG	CACTGTGTTGGCGTACAGGT
**CHIP**	ANGPTL4-promoter-HRE1	GACGGTGACCATCTGAACCT	TCCAAGCCAGCTCATTCTCT
	ANGPTL4-promoter-HRE3	GGCTG GTCTG GAAGT CTTG	GGGCGGAGGAGTCTTGG
